# Epithelial–mesenchymal plasticity: emerging parallels between tissue morphogenesis and cancer metastasis

**DOI:** 10.1098/rstb.2020.0087

**Published:** 2020-08-24

**Authors:** Andrew T. Plygawko, Shohei Kan, Kyra Campbell

**Affiliations:** Department of Biomedical Science and Bateson Centre, University of Sheffield, Sheffield S10 2TN, UK

**Keywords:** tissue morphogenesis, epithelial–mesenchymal transition, mesenchymal–epithelial transition, cancer metastasis, cell plasticity, collective migration

## Abstract

Many cells possess epithelial–mesenchymal plasticity (EMP), which allows them to shift reversibly between adherent, static and more detached, migratory states. These changes in cell behaviour are driven by the programmes of epithelial–mesenchymal transition (EMT) and mesenchymal–epithelial transition (MET), both of which play vital roles during normal development and tissue homeostasis. However, the aberrant activation of these processes can also drive distinct stages of cancer progression, including tumour invasiveness, cell dissemination and metastatic colonization and outgrowth. This review examines emerging common themes underlying EMP during tissue morphogenesis and malignant progression, such as the context dependence of EMT transcription factors, a central role for partial EMTs and the nonlinear relationship between EMT and MET.

This article is part of a discussion meeting issue ‘Contemporary morphogenesis'.

## 1. Introduction

**Figure 1.**
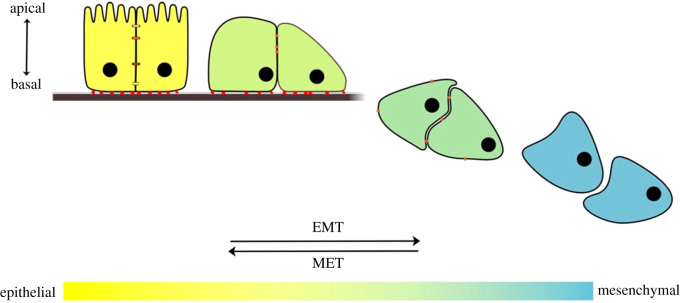
EMT and MET mediate dynamic and reversible changes along a spectrum of intermediate epithelial–mesenchymal phenotypes. Mature epithelial cells (yellow) are characterized by apicobasal polarity, lateral cell–cell junctions such as adherens junctions, gap junctions or tight junctions, and adhesion to a basement membrane (brown). Mesenchymal cells (blue) exhibit front–rear polarity, lack cell–cell adhesions and migrate individually following detachment from the basement membrane. Cells with intermediate phenotypes, induced via partial EMT, can simultaneously possess both mesenchymal and epithelial features, and often migrate as a collective owing to the retention of intercellular adhesion. This diagram acts as an exemplar for the continuum of EMT states, but will differ between tissue contexts *in vivo* depending on the maturity of epithelial cells, junctional arrangements, basal adhesion and modulation of the EMT programme.

A key feature of tissue morphogenesis is the ability of cells to rapidly and reversibly change their phenotype. This is termed cell plasticity and is exemplified by the shift of polarized epithelial cells to an unpolarized migratory mesenchymal state, and vice versa (figure 1). This epithelial–mesenchymal plasticity (EMP) is underpinned by the processes of epithelial–mesenchymal transition (EMT) and mesenchymal–epithelial transition (MET). Both EMTs and METs are crucial for the migration and organization of many cell types into tissues and organs during embryogenesis [1]. They also play key roles in homeostatic contexts, such as during wound healing [2] and tissue renewal [3]. However, when activated inappropriately these processes can contribute to the progression of many diseases, including cancer and fibrosis.

Cancer metastasis is a complex, multistage process, and there is increasing evidence suggesting that cancer cells co-opt this plasticity, critical to healthy development, to accomplish several of these steps. EMTs enable the escape of cells from primary tumours, and their dissemination throughout the body, and can also confer some degree of stemness on cancer cells [4]. Upon their arrival at a distant site, METs have been shown to promote the overt outgrowth of secondary metastases [5]. Metastasis remains the most deadly phase in the malignant progression of a tumour, as well as the most poorly understood [6]. Therefore, increasing our understanding of the cellular and molecular mechanisms underlying epithelial plasticity during development, and investigating parallels with cancer processes, will likely aid in the identification of novel prognostic and therapeutic markers. This review will discuss the parallels between EMP during tissue morphogenesis and in cancer progression, and highlight how developmental systems can be a window into this aspect of pathogenesis.

## 2. Principles of epithelial plasticity

During development, mature epithelial cells exist on a spectrum from cells possessing only apicobasal polarity and nascent junctions, to highly differentiated cells with elaborate cell–cell junctions and specialized apical features, such as brush borders and cilia [7]. Epithelial cell–cell adhesion is often mediated through intercellular junctions comprising proteins such as E-cadherin and ZO-1, providing a method for signalling between epithelial cells, as well as a barrier necessary for tissue or organ function [8]. Apicobasal polarity is required for asymmetry of function, such as absorption and secretion, and is defined by the mutually exclusive localization of multiple protein complexes at the apical and lateral domains of the cell [9]. The specific arrangement and composition of these junctions and polarity complexes differs between cell type and species, resulting in a diversity of function between different tissues. The adhesion of mature epithelial cells to a basement membrane provides additional mechanical support to the tissue, and allows further signalling to occur to dictate cell function, such as through the basally localized integrin complexes [10].

By contrast, mesenchymal cells lack stable cell–cell adhesion and apical–basal polarity, instead adopting a front–rear polarity. These properties, in addition to differences in cytoskeletal organization and interaction with the extracellular matrix, confer a greater migratory capacity on these cells than their epithelial counterparts [11].

EMT was first identified as a process through which cells that are born far from their final destination are able to delaminate, migrate and populate different regions of the embryo [12]. It was only with the later discovery of the EMT-inducing transcription factor Slug that EMT was proposed to play a role in pathogenesis, based in part on the parallels between delamination and tissue escape in the chick mesoderm and cancer metastasis [13]. The Slug-related transcription factor Snail was later shown to be activated in dedifferentiated carcinomas, inducing a cellular transition similar to that described previously in the embryo [14].

Since then, a number of evolutionarily conserved transcription factors have been found to induce EMT, including other genes in the Snail family, zinc-finger E-box-binding (ZEB) family and basic helix–loop–helix (bHLH) family. These encompass the core EMT transcription factors (EMT-TFs), which include Snail/Slug, ZEB1/ZEB2 and Twist1, respectively [11]. Many EMT-TFs were first identified for their role in tissue morphogenesis. For example, Snail and Twist were initially characterized as key regulators of *Drosophila* gastrulation [15,16], ZEB1 as a transcriptional regulator enriched in mesodermal regions following gastrulation in chick embryos [17], and mouse Prrx1 mutants were first analysed in the context of skeletal formation deriving from the cranial neural crest (NC) and mesoderm [18]. These transcription factors act by downregulating the epithelial characteristics of cell–cell adhesion and apicobasal polarity, and by enhancing the migratory capacity of the resulting mesenchymal cells. The reduction in intercellular adhesion is perhaps best represented by the ability of most EMT-TFs to repress the expression of E-cadherin, disrupting the adherens junctions [14,19]. Both Snail and ZEB1 are also able to suppress the expression of the apically localized polarity protein Crumbs3 and its orthologues, providing a conserved mechanistic link between transcription factor activation and the disassembly of apicobasal polarity as well as adherens junctions [20].

These transcription factors, while all possessing the capacity to induce EMT in specific circumstances, differ in their domain structures, mechanisms of action and regulation, resulting in diverse and non-overlapping functions and expression patterns in development [21]. An example is seen during *Drosophila* gastrulation, where the EMT-TF Snail induces EMT in the mesoderm by disrupting adherens junctions through the repression of E-cadherin transcription [22,23]. By contrast, the EMT underlying endoderm morphogenesis does not depend on Snail activity [24,25], instead relying on the GATA factor Serpent [26]. Furthermore, Serpent does not repress E-cadherin, which remains transcriptionally active in the endoderm, instead disrupting apicobasal polarity and adherens junctions through the direct repression of *crumbs* [26].

Differing spatio-temporal expression patterns and functions of EMT-TFs are also observed during EMT and migration of neural crest (NC) cell populations during nervous system development in vertebrates [27]. In *Xenopus*, the first cephalic NC cells express Twist and migrate extensively to the ventral side of the head to form cartilage [28]. However, the peripheral nervous system is formed in part through the migration of a second cephalic NC population to the dorsal side of the head, which instead relies on Snail2 expression [29]. In chick, the trunk NC cells undergo EMT from the dorsal neural tube all along the anteroposterior axis. At any given position along this axis, EMT occurs for 48 h, resulting in two populations of trunk NC cells. During the first 24 h, an initial NC population migrates to form the peripheral nervous system, and expresses both Snail2 and ZEB2. Following this, Snail2 is no longer expressed in the neural tube, resulting in the delamination and migration of a second population which expresses only ZEB2 [30], later differentiating into melanocytes. These two populations take different migratory paths, with the first migrating through the paraxial mesoderm and the second following a dorsal trajectory under the endoderm, and also migrate at differing cell densities. The EMT-TF expression patterns of these two trunk NC populations contrast with the chick cephalic NC, where the combined action of Snail2 and Ets1 promote EMT and migration [31,32].

Many EMT-TFs first identified for their role in development have been found to be reactivated at the invasive front of carcinomas [33–35] and are required for metastasis. These EMT-TFs not only act through similar cellular and molecular mechanisms to those deployed during development, but also display a level of context dependence that has only recently been fully appreciated in the study of cancer. For example, the ability of cells in a pancreatic cancer model to undergo metastasis and form secondary tumours in the absence of Snail was used as evidence that EMTs are not required for metastasis [36]. However, a later study showed that, while Snail is indeed dispensable for metastasis in this pancreatic cancer model, the depletion of ZEB1 strongly affected EMP and metastatic potential [37]. Furthermore, endogenous Snail has been shown to be important for metastasis in the MMTV-PyMT-driven breast cancer model and is associated with mesenchymal features in tumour cells [33]. Taken together, these studies demonstrate that, as in development, the EMT-TF involved is dependent upon the tissue context.

The environmental and epigenetic circumstances within which a tumour forms, as well as the type of epithelium, can also affect the propensity of tumour cells to undergo EMT. This is exemplified by recent findings that tumorigenesis induced through the same oncogenic insults resulted in epithelial tumours in the skin epidermis, but tumours undergoing EMT in the hair follicle [38]. Analysis of chromatin state and gene transcription in both of these tissues, and in their tumorigenic counterparts, revealed that the hair follicle lineage was primed to express multiple TFs associated with EMT induction [38]. Several of these EMT-TFs, such as Runx1 and Tbx1, have established roles in hair follicle stem cell homeostasis [39,40], suggesting that their expression or priming in non-tumorigenic cells is linked to developmental functions that are then aberrantly used by tumour cells. When this observed context-specificity for EMT induction is paired with the fact that the EMT-TFs employed differ between developmental systems, it raises the possibility that the mechanisms for EMT and MET used during cancer progression in a specific tissue may mirror those used by that tissue during development.

## 3. The spectrum of EMT in development and cancer

While initial studies of EMT considered it to be a binary process during which a mature epithelial cell transitions to a fully mesenchymal phenotype, an increasing number of studies have demonstrated that EMT can occur to differing extents, resulting in a spectrum of cell types coexpressing a variety of epithelial and mesenchymal characteristics [2,7,11]. These so-called partial EMTs are prevalent in development, and can arise from two considerations. The first is that the complexity and maturity of the initial epithelium upon EMT onset determines to what extent a full EMT would need to occur before cells were considered fully mesenchymal; the second is that the EMT programme can be undertaken incompletely [7]. The incomplete transition to a mesenchymal cell occurs in numerous developmental systems, and often results in the maintenance of partial cell–cell junctions (figure 1). This simultaneous adoption of migratory capacity while maintaining intercellular adhesion is associated with the collective migration of many cell populations [7].

**Figure 2.**
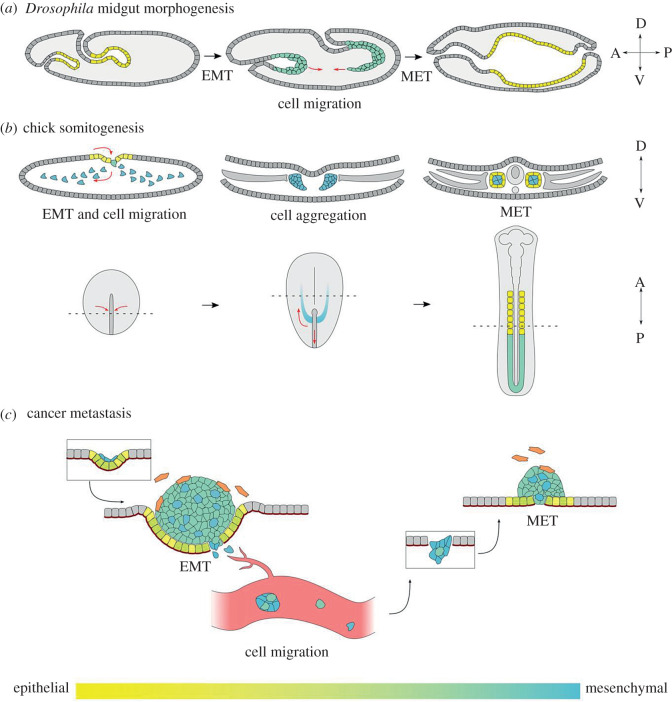
Successive rounds of EMT and MET take place in development and cancer. (*a,b*) Several developmental examples exist in which both EMT and MET of the same cell population can be studied. (*a*) *Drosophila* embryonic midgut morphogenesis*.* Midgut cells at both the anterior and posterior of the embryo are initially epithelial (yellow). These cells then undergo a partial EMT (green), which facilitates their collective migration through the embryo. The majority of these migrating cells subsequently undergo MET to form a contiguous midgut epithelium. The compass indicates the anterior–posterior and dorsal–ventral axes. (*b*) Chick somitogenesis. The upper row of diagrams shows transverse cross sections. Sectioned regions are indicated by dotted lines on the lower row of diagrams, which depict a dorsal view of the embryo. Epithelial cells (yellow) at the primitive streak first undergo EMT to form mesenchymal mesoderm progenitors (blue). The paraxial mesoderm cells (blue) then undergo migration towards the anterior of the embryo to form the presomitic mesoderm (green). The presomitic mesoderm undergoes MET during segmentation to form somites. Mature somites consist of epithelial cells (yellow) enclosing mesenchymal cells (blue). (*c*) EMT and MET underlie distinct stages of tumour metastasis. Cancerous epithelial cells proliferate and can undergo partial or full EMT to form heterogeneous tumours (coloured) within an otherwise non-cancerous tissue (grey). Tumours also recruit other, non-cancerous cells to facilitate growth and invasion, such as tumour-associated fibroblasts (orange). Migratory cancer cells can escape the tissue via individual or collective migration and enter the circulatory system as individual cells or as clusters, dependent on EMT state. Metastatic colonization of secondary sites requires re-epithelialization of the tumour cells through MET, resulting in the formation of secondary tumours.

A developmental example of a partial EMT resulting in collective migration of a heterogeneous cell type is the formation of the *Drosophila* embryonic midgut (figure 2*a*). During embryogenesis, endodermal epithelial populations at the anterior and posterior poles, termed the anterior and posterior midgut primordia, undergo an EMT which facilitates their collective migration through the embryo [24]. This process is accompanied by the differentiation of endodermal cells into three distinct populations that migrate in coordination with one another along the underlying mesoderm [25,41–43]. The largest of these three populations are the principal midgut epithelial cells (PMECs), which later undergo MET to form a monolayered epithelium, while the other two populations retain mesenchymal morphology [41]. The EMT undertaken prior to collective migration of the *Drosophila* posterior midgut is also considered partial, as E-cadherin is not wholly repressed and remains partially localized to the plasma membrane in a punctate fashion [26]. These punctae are required for the migration of this otherwise-mesenchymal cell population, as depletion of E-cadherin results in the loss of cell–cell cohesion during collective movement [43]. This partial EMT is induced through the activation of the GATA transcription factor Serpent, which drives a loss of apicobasal polarity and fragmentation of adherens junctions through the direct repression of *crumbs* [26]. This role for Serpent has been demonstrated to be conserved by its mammalian orthologues GATA4 and GATA6 in canine kidney cells [26], and GATA6 upregulation has been linked to tumorigenesis in multiple tissues [44,45]. However, GATA6 has conversely been found to suppress EMT in pancreatic cancer cells, suggesting divergent roles in different tissue contexts and underscoring the importance of studying gene function in multiple developmental systems [46].

A common aspect linking developmental models for partial EMT and collective migration is the maintenance of E-cadherin expression. Though EMT-TFs are capable of repressing E-cadherin [14,19], and its loss is considered a hallmark of the mesenchymal state, it has become increasingly clear that the spectrum of EMT states begets a spectrum of E-cadherin-mediated adhesion states. Indeed, in the migration of both the posterior midgut and the border cell cluster in the *Drosophila* egg chamber, the presence of E-cadherin is required for maintenance of migratory cell clusters, as border cell-specific depletion results in cluster disassembly, and the disruption of E-cadherin in migrating posterior midguts results in the detachment of otherwise-collective mesenchymal cells from one another [43,47]. The expression and functional relevance of E-cadherin in cells undergoing a partial EMT can also be observed during vertebrate development, as the collective migration of the zebrafish mesendoderm and the *Xenopus* cranial NC both rely upon continued intercellular adhesion conferred by E-cadherin [48,49]. Furthermore, though the downregulation of E-cadherin and expression of N-cadherin is required to establish collective migration in the *Xenopus* cranial NC, residual levels of E-cadherin protein still remain on the membrane of these cells, indicating that its repression is not absolute [50]. This low level of E-cadherin expression is still required for migration of cranial NC cells, though its knockdown does not affect cell–cell adhesion within the collective, potentially reflecting a function in adhesion of the cranial NC to the adjacent placode cells [51]. Moreover, an examination of the expression pattern of E-cadherin in mouse and chick cranial NC cells showed that E-cadherin protein perdures during delamination and early migration, though the functional relevance of this has yet to be investigated [30,52]. These data suggest that the maintenance of E-cadherin expression following induction of EMT programmes may be more widespread than previously envisaged.

The initial assessment of EMT as a binary transition between two end states resulted in controversy regarding the role of EMT in cancer progression, as there was insufficient evidence from clinical samples for such a full transition [53]. However, mirroring studies in development, it has become increasingly clear that EMT in cancer is often partial, resulting in tumour cells simultaneously adopting features of both epithelial and mesenchymal cells [4].

The advent of single-cell technologies has allowed partial EMTs to be identified in unprecedented resolution. The first such example found that induced tumours in the mouse hair follicle—previously shown to exhibit properties of EMT [38]—contained several different subpopulations of cells undergoing varying levels of EMT [54]. These populations were spatially distinct within the tumour mass, and populations undergoing a partial EMT possessed a greater metastatic potential than their epithelial or mesenchymal counterparts, recapitulating *in vitro* data on the clinical relevance of incomplete transitions between these two states [54]. Mass cytometry of high-grade serous ovarian cancer tissue identified cell populations simultaneously expressing both E-cadherin and the mesenchymal marker vimentin, with some of these populations being associated with metastasis following patient relapse [55], while mass cytometric studies of lung cancer cell cultures identified a spectrum of EMT profiles which could also map to cell populations within patient tissue [56]. These *in vitro* and *in vivo* analyses, when considered cumulatively, suggest a strong association between partial EMT states and greater metastatic capacity and the resultant prognosis in a multitude of different carcinomas [57].

The increasing evidence for the adoption of partial EMT states by multiple tumour cells has been accompanied by a re-evaluation of the role of E-cadherin in invasion and metastasis. Early studies found that E-cadherin expression was often ablated in breast cancer [58], and was depleted in cells at the invasive front in colorectal cancer tissues [59]. Repression of E-cadherin was further correlated with increased tumour migration and metastatic capacity *in vitro* and in a mouse breast cancer model [60,61], suggesting a functional link between E-cadherin downregulation and cancer progression. However, this perturbation of E-cadherin does not appear to be universally required for metastasis. An elegant study undertaken using MMTV-PyMT mice and organoids showed that, though loss of E-cadherin increased the invasive potential of tumour cells in three-dimensional invasion assays, transplantation of E-cadherin mutant tumour organoids into mice resulted in an almost-complete ablation of metastatic potential compared with their wild-type tumour counterparts [62]. E-cadherin mutant organoids also formed significantly fewer macrometastases than wild-type when injected directly into tail veins, indicating that this discrepancy in colonization was independent of their ability to escape from the primary tumour. Consistent with this implication, E-cadherin mutant cells exhibited increased proportions of apoptosis mediated by reactive oxygen species, suggesting a novel function for E-cadherin beyond its well-established role in adhesion [62].

Other studies of E-cadherin in tumour invasion and metastasis have identified more nuanced relationships. In a mouse pancreatic ductal adenocarcinoma model, genetic manipulation of the E-cadherin interacting partner p120-catenin was used to demonstrate that E-cadherin expression and epithelial status were associated primarily with liver metastases, whereas disruption of E-cadherin shifted metastatic colonization to the lungs [63]. The authors suggest that this difference stems from an inability for cells in a fully mesenchymal state to metastasize to the liver, whereas cells having undergone partial EMT are capable of forming both liver and lung metastases. A separate examination of EMT states in a mouse pancreatic cancer model revealed that partial EMT was mostly driven by a mechanism that is independent of EMT-TF-mediated transcriptional repression, whereby E-cadherin was internalized and sequestered in vesicles through endocytosis [64]. The same mechanism was also identified in both colorectal and breast cancer cell lines, indicating it may be a widespread programme activated during tumour dissemination, though it has yet to be identified in breast or colorectal tumours *in vivo* [64]*.* This partial EMT induction through E-cadherin relocalization has numerous parallels with tissue morphogenesis. During both *Drosophila* posterior midgut migration and zebrafish epiboly, E-cadherin is endocytosed as opposed to transcriptionally repressed, limiting its presence on the plasma membrane [43,65]. Interestingly, during sea urchin mesoderm formation, Snail activity is required for both the endocytosis of E-cadherin and for its transcriptional repression, indicating that these two modes of EMT induction need not be mutually exclusive in all circumstances [66]. It is possible that, as in sea urchin embryogenesis, future investigations will identify cancers in which both E-cadherin relocalization and transcriptional repression are deployed, further adding to the spectrum of partial EMTs identified during tumorigenesis.

Given the numerous developmental models of partial EMT that are twinned with collective migration, it is unsurprising that many cancer cells migrate as a collective [67]. Collective cell migration has been linked to the retention of certain epithelial characteristics such as intercellular adhesion in cancer using both three-dimensional cell culture and MMTV-PyMT mouse models, as it has been demonstrated that cells expressing epithelial markers such as cytokeratin 14 and E-cadherin act as leader cells during this migration [68,69]. Additionally, in a *Drosophila* metastatic tumour model in which EMT is activated, cells maintained E-cadherin expression and escaped from the primary tissue as a collective [70]. However these studies, and also studies of clinical samples exhibiting collective migration [71,72], rely on sample fixation and therefore do not allow the examination of migratory speed, heterogeneity or regulation of collective tumour cells *in vivo*. Furthermore, there are currently limited models for murine intravital imaging capable of consistently detecting tumour collective migration [73], complicating analysis of migratory cell dynamics. The use of developmental models may circumvent this issue, owing to their ease of imaging and genetic tractability. Given that clusters of circulating tumour cells (CTCs) have a far greater potential for seeding secondary metastases than single cells [74], understanding the dynamics of their movement and the mechanisms mediating their adhesion may aid the development of treatments targeted at cluster dissociation, as will be discussed in the next section.

## 4. Partial EMT, adhesion and circulating tumour cells

Following escape from the primary tissue, migrating tumour cells can enter the circulatory system to reach distant sites in the body. When present in the bloodstream these cells are termed CTCs, and represent an intermediate step between primary tumour dissemination and metastatic colonization (figure 2*c*). While examination of blood from breast cancer patients found that the majority of CTCs migrate individually, a small proportion were capable of migrating as clusters of cells expressing a combination of epithelial and mesenchymal markers, indicative of partial EMT [75]. Multiple lines of analysis suggest that clustered CTCs escape the primary tumour as a collective as opposed to aggregating during circulation, providing further support for the role of partial EMT in forming CTC clusters [76]. The formation of CTC clusters is of clinical interest given that clusters have a significantly greater metastatic potential than single CTCs in spite of their rarity [74], owing in part to their resistance to apoptosis [62,74], and are accordingly associated with poor prognosis in multiple cancers [77].

Understanding CTC adhesion could prove informative for the development of treatments aiming to limit metastasis through cluster dissociation. Encouragingly, several adhesion molecules have already been identified in these clusters, many of which are associated with epithelial cell fate. E-cadherin has been identified at the point of cell–cell contact within these clusters in a mouse model of pancreatic cancer [64], and is also expressed in CTC clusters in a mouse breast cancer model [62]. Though cells lacking E-cadherin in the pancreatic cancer model migrated individually, breast cancer CTC clusters could be identified that lacked E-cadherin expression, indicating that its depletion is not necessarily sufficient to block CTC clustering [62,64]. Interestingly, a recent study of a squamous cell carcinoma model found that claudin-11 expression was correlated with collective migration and CTC cluster formation, and its expression was driven in this context by the EMT-TF Snail, providing an additional mechanistic link between EMT onset and poor prognosis [78]. Single-cell RNA sequencing of patient-derived breast cancer CTC clusters also found that the adherens junction- and desmosome-associated protein plakoglobin was upregulated in clustered cells, and that its knockdown resulted in cluster dissociation [74].

The adhesion glycoprotein CD44 is also expressed in breast cancer CTC clusters, and its depletion blocked the aggregation of cells and was associated with decreased metastatic potential following injection of treated cells into mice [79]. The identification of upregulated CD44 was particularly interesting given CD44 is commonly used as a marker for stemness properties in cancer cells [80]. During development, intercellular adhesion is required for the maintenance of pluripotency in embryonic stem cell populations, and its disruption leads to the loss of stemness [81]. In a fascinating parallel to this developmental context, methylome profiling of breast CTCs found that clusters, but not single cells, exhibited hypomethylation of binding sites for numerous stem-associated transcription factors [82]. Furthermore, the disruption of intercellular adhesion in these clusters resulted in a reversal of hypomethylation at these sites, effectively reversing the stem-associated profile in the constituent cells. It is therefore possible that the further identification of developmentally relevant cell adhesion molecules in stem cells will provide therapeutic insights into the treatment of CTC clusters from multiple carcinomas in the future.

Beyond the metastatic advantage provided by their resistance to apoptosis [62,74], it is becoming increasingly clear that the heterogeneity of CTC clusters enhances their potential to colonize a distant site. Tumour cells within each cluster can associate with non-tumour cells that aid in their metastasis. For example, lung cancer CTCs can incorporate tumour-associated fibroblasts into their cluster, improving both the viability of the tumour cells and the metastatic potential, likely through microenvironmental alterations made by the fibroblasts to produce a site amenable to further tumour growth [83]. Two recent studies of CTC cluster composition identified additional interactions with two populations of myeloid-derived immune cells, both of which promoted metastasis through signalling between tumour and immune cells to induce proliferation [84,85]. Though it remains unproven, it is tempting to speculate that heterogeneity may exist within the CTC cluster population with respect to stemness traits. The adhesion between heterogeneous cell populations within CTC clusters need not necessarily rely on the same factors as those ensuring cohesion between CTCs, as Vcam1 mutation disrupts the CTC–neurophil interaction but not the interaction of CTC clusters themselves [85]. These CTC clusters, and their associated non-tumour cells, therefore migrate as a heterogeneous population of cells with variations in fate, EMT state and potentially stemness. It is likely that future studies turning to developmental models of heterogeneous collective migration could identify further factors required for adhesion and concerted migration of CTC clusters, presenting potential targets for prognostic and therapeutic use.

## 5. Understanding the relationship between EMT and MET

While EMT can promote the dissemination of cancer cells from primary tumours, an increasing number of studies point to a key role for MET and the re-epithelialization of mesenchymal cells in the growth of secondary tumours (figure 2*c*). Considering that MET is often described as the ‘reverse of EMT’, and EMT has become a target of prime interest for anti-cancer therapy [86], this raises an important question: is inhibiting or removing EMT-inducing factors the same as promoting MET? If so, this type of approach could have catastrophic side-effects, driving metastatic progression in patients with cells that have already disseminated from primary tumours.

The mechanisms driving MET are far less well understood than those underlying EMT, and it remains unclear how the mechanisms driving these processes relate to one another. Rather than stimulating MET through activation of a specific transcription factor or signalling pathway, in many *in vitro* and *in vivo* cancer studies MET is activated through the turning off of an EMT-inducing signal such as transforming growth factor β (TGF-β) [56,87], or through the downregulation of an EMT-TF [88,89].

In line with this, in the few systems where MET has been studied during tissue morphogenesis, EMT-TFs need to be downregulated for MET to take place. Somite formation relies on a cycle of EMT and MET, where presumptive paraxial mesoderm cells transition to a mesenchymal state as they move through the primitive streak, and later re-epithelialize as blocks of tissues segregated from the anterior presomitic mesoderm (figure 2*b*). Paraxial mesoderm cells maintain high levels of *snail1* and *snail2* as they migrate and form the presomitic mesoderm [90]. However, both genes are downregulated as the cells move from regions with high levels of fibroblast growth factor (FGF) to those with low levels, and this coincides with their acquisition of epithelial characteristics. It was demonstrated in chick that the overexpression of Snail2 blocks MET, with the cells failing to express *paraxis*, a gene thought to be critical for their re-epithelialization [90]. When considering the cycle of EMT and MET that underlies embryonic midgut formation in *Drosophila*, a similar relationship is seen. Endogenous Serpent expression is downregulated in endoderm cells just after they undergo EMT and initiate migration. When Serpent expression is maintained in the cells, they never undergo MET and remain mesenchymal throughout embryogenesis [26]. These results support a central role for the downregulation of EMT-TFs in order for MET to take place.

However, EMT-TF downregulation does not appear to be sufficient for MET in these developmental contexts, which also rely on interactions with neighbouring tissues. Removal of the surface ectoderm, which lies adjacent to the somites, results in a failure of the cells to undergo MET [91,92]. This is not likely to be due to a failure in the downregulation of *snail* genes, as this occurs downstream of changes in FGF levels, which are independent of interactions with the ectoderm. Furthermore, the initial expression of *paraxis* has been observed in explants lacking ectoderm, suggesting that Snail is indeed downregulated [93]. Instead, the ectoderm is required for the deposition of a fibronectin matrix, which is absolutely critical for the re-epithelialization of the somites [93,94]. Similarly, MET in the *Drosophila* endoderm relies on interactions with the underlying mesoderm [25], although whether this is required for downregulating Serpent, or for providing additional cues, remains to be explored.

Tissue–tissue interactions also play a role in the MET that underlies the transition of the metanephric mesenchyme into epithelial tubes, during morphogenesis of the vertebrate kidney. MET in the metanephric mesenchyme relies on factors secreted from the neighbouring ureteric bud, such as Wnt9b and Wnt4 [95,96]. In response to these signals, subpopulations of the metanephric mesenchyme first condense into pretubular aggregates, which then undergo MET, polarizing and forming an intracellular lumen. Although it is not clear whether any EMT-TFs are downregulated at this stage, it is interesting to note that the subpopulation of the metanephric mesenchyme that undergoes EMT is a self-renewing, multipotent Six2-expressing progenitor population [97]. Six2 is essential for maintaining the progenitor state, and suppresses epithelial differentiation. Concordantly, Six2 loss results in premature epithelialization of the metanephric mesenchyme in the embryonic kidney, while ectopic expression of Six2 represses the differentiation of mesenchymal cells into epithelia in a kidney culture model [97]. Intriguingly, a more recent study of Six2 in breast cancer found that Six2 expression is associated with greater metastatic potential owing to both its induction of ZEB2 expression and its repression of E-cadherin transcription through promoter methylation [98]. It would be interesting to explore if this mechanism also underlies the ability of Six2 to block MET in the metanephric mesenchyme.

Interactions between mesenchymal cells and contacting tissues are also important factors affecting MET during cancer progression. It has long been recognized that while the properties of a single CTC, or a CTC cluster, are important determinants in whether it will succeed in forming a metastatic outgrowth, the properties of the tissue in which CTCs attempt colonization are equally important [99]. The outcome of metastasis is dependent on cross-talk between tumour cells and receptive tissues, and the ability to induce MET may form part of this discourse. Crucially, it is becoming increasingly clear that tumour cells are themselves capable of altering these receptive tissues to their benefit. An elegant example of this was recently demonstrated in a mouse model where the so-called ‘metastatic initiating cells' (MICs) were isolated from the mouse breast cancer MMTV-PyMT model, tail-vein injected, and the mechanisms underlying the formation of resulting lung metastases investigated. It was found that colonization of the lung relies on a cross-talk between the mesenchymal MICs and the lung stroma. The MICs induced activation of the lung fibroblasts through secretion of the extracellular protein thrombospondin 2, and this in turn drove MET in the MIC cells through the inhibition of TGF-β signalling [98].

Bone metastases in mouse models also demonstrate tumour cell cross-talk with their receptive tissues, during which MET is both induced in the tumour cells and required for the development of secondary growths [100]. The bone vascular niche, normally populated by haematopoietic progenitor cells (HPCs) and haematopoietic stem cells (HSCs), represents a common site for secondary metastases for breast cancer and prostate cancer [101]. Evidence from mice suggests that pancreatic MICs use many of the same molecular pathways normally used by HSCs to localize to the bone vascular niche [101]. Of note is the role of E-selectin, which typically mediates the capture of circulating HSCs and HPCs into the bone marrow in a process called homing [102,103]. Metastatic cells are able to repurpose this E-selectin binding as a means to promote metastasis; in mouse models, metastatic breast cancer cells were shown to undergo MET upon binding with E-selectin expressed on the vascular endothelium [100]. E-selectin-induced MET did not affect the RNA expression of master transcriptional regulators of EMT, such as Snail1/2, Twist1/2 or ZEB1/2, instead appearing to affect EMT proteins such as N-cadherin and Slug at the post-translational level. The observations in this study point towards an MET programme that is not the binary opposite of traditional EMT programmes, which the authors suggest is a non-canonical MET [100].

The poor characterization of molecular drivers of MET during metastatic colonization, and observations from studies of metastasis such as those discussed above [100], raise the question of what actually constitutes a canonical MET. There are some suggestions from developmental contexts that the mechanisms driving MET may be quite distinct from those that were disassembled during EMT in the initial epithelium. For example, in zebrafish embryos, repolarization of the somites during MET is dependent on integrin α5 and the extracellular matrix protein fibronectin [104]. However, neither protein appears to play major roles in maintaining the epithelial state during earlier stages of embryogenesis, as a deficiency of either fibronectin or integrin α5 does not affect axis elongation [104]. In *Drosophila,* Crumbs is a key regulator of apicobasal polarity and adherens junction formation in the first epithelium that forms in the embryo [105]. The transcriptional repression of *crumbs* is the central mechanism by which apicobasal polarity is disassembled during the EMT that underlies endoderm formation [26]. However, Crumbs and other apicobasal polarity proteins such as Stardust, the *Drosophila* orthologue of Pals1, are never re-expressed in the midgut, the sole endoderm derivative in *Drosophila* [106]. Furthermore, though the mechanisms underlying midgut MET in the embryo are poorly understood, it has recently been demonstrated that the cells in the adult midgut polarize using an alternative mechanism, which does not appear to require many of the factors required for polarity in the embryonic ectoderm—the epithelium from which it is derived—including Par-3, Par-6, atypical protein kinase C, Scribbled, Discs large and Lethal giant larvae [107]. Additionally, while E-cadherin repression is central to EMT as cells move through the primitive streak in vertebrates; it is not one of the key factors involved in the MET that drives formation of the nephric vesicles from the metanephric mesenchyme during kidney morphogenesis. Instead, afadin, a nectin adaptor protein, is required for de novo lumen formation *in vivo,* acting upstream of the recruitment and/or stabilization of the predominant cadherin, R-cadherin, during repolarization of renal vesicles [108]. Thus, in the few developmental systems where it has been examined in detail, repolarization and re-epithelialization appear to be driven by mechanisms distinct from those underlying the original transition towards a mesenchymal state.

**Figure 3.**
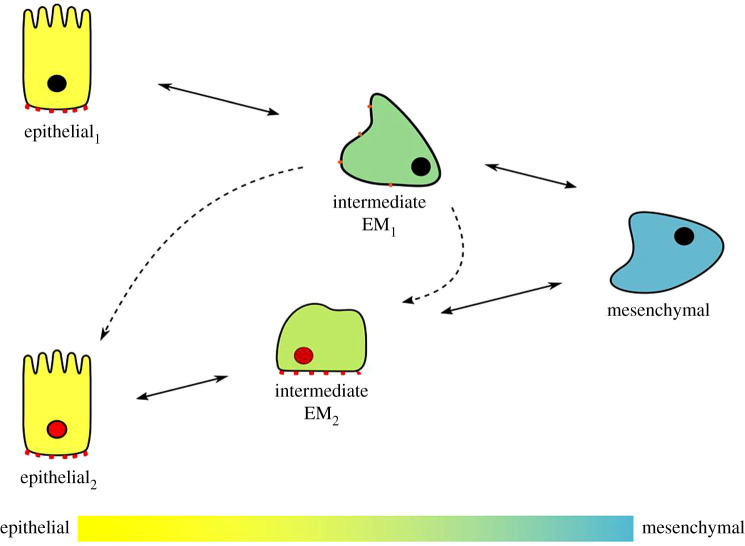
EMP occurs along branched trajectories to produce divergent cell fates. The extent to which EMT is activated, and the nature of the epithelial cell in which EMT is induced, results in a broad spectrum of partial EMT states. However, given that MET is not simply the reversal of the EMT process, the end result of this programme is not a reversion to the initial epithelial state (yellow with black nucleus) but the adoption of a second, distinct epithelial cell fate (yellow with red nucleus). It is worth noting that, though the arrows in this diagram indicate a complete progression through to a fully mesenchymal state prior to MET, it is also possible for cells exhibiting a partial EMT to undergo MET towards a second epithelial state, effectively bypassing full EMT.

Recent studies examining EMP during cancer progression also suggest that EMT and MET are not simply the reverse of one another. Single-cell cytometry of lung cancer samples demonstrated that cells that undergo sequential EMT and MET, induced by TGF-β treatment and withdrawal, respectively, undergo transitions following distinct branched trajectories that do not mirror each other [56]. Moreover, successive EMT/MET in epithelial cancers appear to promote re-epithelialization through alternative pathways, such that the cells do not re-acquire traits of their previous epithelial cell state, and epithelial tumour cells after EMT/MET often demonstrate greater tumorigenic potential. For example, studies of patient tissue found that all metastatic tumours originating from ductal carcinoma expressed or re-expressed E-cadherin, irrespective of the E-cadherin status of the primary breast tumour, demonstrating differential expression of E-cadherin in epithelial tumour cells following MET compared with before EMT onset [109]. Additionally, RNA-seq of *in vivo* models of bone metastases showed that following MET, specific subsets of genes that promoted establishment of a metastatic niche were upregulated [87]. Furthermore, re-epithelialized prostate cancer cell lines transformed by transient induction of Snail exhibited a specific transcriptional signature which was lacking in untreated epithelial cells [89]. The clusters of genes differentially expressed in the re-epithelialized cells were associated with poor prognosis in various cancers and castrate resistance in prostate cancer, illustrating the dramatic difference in transcription profile and metastatic potential between epithelial cancer cells pre- and post-MET [89]. These distinctions between cell state following EMT/MET and cell state in epithelial cells prior to EMT challenge the paradigm that EMT and MET are reverse processes, and may be indicative of different regulatory mechanisms (figure 3). Taken together, this suggests that rather than pursuing a strategy of blocking EMT through targeting of an EMT-TF or its cofactor, which may prime already-disseminated CTCs to undergo MET in response to local cues in the environment, it may be better to focus on identifying mechanisms unique to either EMT or MET, as these could present more specific targets for therapeutic intervention.

## 6. Conclusion and future perspectives

Since the initial characterizations of EMP in the developing embryo [12], several principles relating to the nature of the underlying transitions, and their relation to one another, have been identified and investigated in both cancer and development. These joint studies have, in recent years, resulted in the emergence of several parallel concepts. Examinations of morphogenesis and pathogenesis have, for example, demonstrated that EMT and MET are not simply transitions between two endpoints, but multifaceted and diverse programmes activated to varying degrees, creating a continuum of partial epithelial and mesenchymal cell states [7]. The increasing diversity of observed EMT states in cancer, coupled with an increasing appreciation of their clinical relevance, necessitates their further interrogation in the pursuit of novel therapies.

Additionally, the fields of cancer research and development appear to be converging—albeit through different lenses—on the concept that EMT and MET are not merely inverse processes. This view, which suggests that MET does not result in reversion to the initial epithelial state but in the adoption of a new and divergent epithelial fate (figure 3), is supported by transcriptional analysis of metastatic cells, as well as morphological and genetic studies during development [56,87,89,104,106,108]. Such a concept may prove therapeutically useful if the gene signatures or requirements specific to cancer MET during metastasis can be identified and disrupted.

However, in spite of these emerging concepts, advances in understanding the epithelial–mesenchymal trajectories followed by tumours—from primary growth to tissue escape to metastasis—are often stymied by the constraints of animal tumour models, from their genetic inflexibility to their difficulty to image over the course of tumour progression [73,110]. Developmental models have now been identified in which EMT, migration and MET can be investigated in the same cell population, enabling the study of how each of these steps relates to each other. Invertebrate and lower vertebrate models in particular are amenable to *in vivo* live imaging at the subcellular resolution, allowing dynamic changes to cell morphology and behaviour to be observed over time [26,43,111]. Additionally, these provide a genetic tractability that allows the intricate dissection of different signals and pathways that play roles in these processes, and the contributions their disruption may make to aberrant behaviours such as tumour formation. It seems advantageous, therefore, for developmental models to be adopted as additional methods for the study of the EMT spectrum and migration, when trying to understand the cellular and molecular dynamics of these processes in tumorigenesis.

Excitingly, the increasing use of single-cell sequencing technology may also help to further bridge the gap between developmental and cancer models. The use of single-cell analysis has contributed significantly to the understanding of the diversity and functional relevance of partial EMT states and MET-associated gene signatures in cancer [54–56,82,87,89,112]. Single-cell methods are also being used to better understand the processes underlying normal development, and to identify novel cell populations and lineages [113]. These studies provide a unique opportunity; if these data from single-cell studies in development and cancer are considered in tandem, it may aid in the identification of developmental systems that are well-suited to act as models for the progression of specific cancer types. This pairing of models with one another could be undertaken on the basis of shared marker expression, gene cluster upregulation or stemness traits, and allow greater overlap and collaboration between the fields of development and cancer research, to their mutual benefit.

## Data accessibility

This article has no additional data.
